# Treatment of solid tumor with autologous natural killer cells in mouse models

**DOI:** 10.1186/2051-1426-2-S3-P23

**Published:** 2014-11-06

**Authors:** Nan-Shih Liao

**Affiliations:** 1 Academia Sinica, Taipei, Taiwan

## 

Cancer immunosurveillance protects the host from outgrowth of tumor cells. Natural killer (NK) cell function positively associates with reduction of cancer risk and with better survival of gastrointestinal stromal tumor patients. We expanded murine NK cells and characterized their anti-tumor activity in vitro and in vivo. Adoptive transfer of the expanded NK cells into tumor-bearing mice reduced tumor burden in B16/OVA and B16/F10 melanoma and Lewis lung carcinoma models. The adoptive transfer treatment enhanced IFN-γ production by splenocytes of the B16/OVA-bearing mice. Moreover, multiple transfers significantly prolonged the survival of mice bearing B16/F10 tumors. These results indicate the potential of autologous NK cell therapy for solid tumors.

